# Challenges and Opportunities of Centrifugal Microfluidics for Extreme Point-of-Care Testing

**DOI:** 10.3390/mi7020032

**Published:** 2016-02-19

**Authors:** Issac J. Michael, Tae-Hyeong Kim, Vijaya Sunkara, Yoon-Kyoung Cho

**Affiliations:** 1 Department of Biomedical Engineering, School of Life Sciences, Ulsan National Institute of Science and Technology (UNIST), 100 Banyeon-ri, Eonyang-eup, Ulju-gun, Ulsan 689-798, Korea; issac@unist.ac.kr (I.J.M.); thkim02@unist.ac.kr (T.-H.K.); vijaya@unist.ac.kr (V.S.); 2 Center for Soft and Living Matter, Institute for Basic Science (IBS), UNIST-gil 50, Ulsan 689-798, Korea

**Keywords:** diagnostics, centrifugal microfluidics, point-of-care, developing countries, clinical chemistry, immunoassays, nucleic acid tests

## Abstract

The advantages offered by centrifugal microfluidic systems have encouraged its rapid adaptation in the fields of *in vitro* diagnostics, clinical chemistry, immunoassays, and nucleic acid tests. Centrifugal microfluidic devices are currently used in both clinical and point-of-care settings. Recent studies have shown that this new diagnostic platform could be potentially used in extreme point-of-care settings like remote villages in the Indian subcontinent and in Africa. Several technological inventions have decentralized diagnostics in developing countries; however, very few microfluidic technologies have been successful in meeting the demand. By identifying the finest difference between the point-of-care testing and extreme point-of-care infrastructure, this review captures the evolving diagnostic needs of developing countries paired with infrastructural challenges with technological hurdles to healthcare delivery in extreme point-of-care settings. In particular, the requirements for making centrifugal diagnostic devices viable in developing countries are discussed based on a detailed analysis of the demands in different clinical settings including the distinctive needs of extreme point-of-care settings.

## 1. Introduction

Centrifugal force-based systems have been used in various biological applications for many years, with the most modern form of this simple technology used even today in clinical laboratories. Centrifugal microfluidic technology combines the benefits of both microfluidics and centrifugal forces in a single device. Rotating the microfluidic disc at various spinning rates induces three different forces on the disc: centrifugal, Coriolis, and Euler forces, each of which can be applied to various microfluidic device operations in an automated manner [1,2,3].

Centrifugal microfluidic technology has been identified as a strong candidate for point-of-care *in vitro* diagnostics (IVD) and has achieved significant commercial success [3,4]. Major multinationals such as Roche and 3M, among others, offer commercial products based on this technology. IVD procedures include clinical chemistry, immunoassays for protein detection, and nucleic acid testing for molecular detection [5]. These IVD procedures involve complex biological assays that usually require a sequence of steps, including sample preparation, incubation, washing, *etc.* All of these steps can be integrated onto a single disc in a centrifugal microfluidic device [4]. The device’s motor can also be programmed to adopt multiple fluid spinning profiles for specific applications. This ensures that the entire device remains compact. Most commercially available centrifugal microfluidic devices have a small footprint and operate as simple sample-to-answer micro-total analytical systems (µTASs). When compared to other microfluidic technologies, centrifugal microfluidic technology has demonstrated many advantages like eliminating the need for multiple pumps, capability to process samples with a wide range of volume and to have pre-stored reagents, and simple and robust operation, which make the centrifugal microfluidic technology suitable for applications in point-of-care testing (POCT).

Extreme point of care testing (EPOCT) is POCT performed in an unfavorable environment characterized by a lack of basic infrastructure such as clean water, dust-free air and surfaces, stable temperatures, uninterrupted power supplies, and other conditions that are the basic requirements for the operation of existing POCT devices [6]. Despite significant advancements achieved in POCT devices, centrifugal microfluidics technology has not yet been considered for use in EPOCT devices.

Present diagnostic tools used in EPOCT are limited to lateral flow strips (LFSs); it is comprised of capture molecules and nanoparticle functionalized on a nitrocellulose membrane that are housed inside a protective plastic casing [7]. LFSs are used both in POCT and EPOCT because of their reasonable cost, easy usage, rapid tests, acceptable sensitivity, and availability for a wide range of diseases [8,9]. Currently, LFSs are widely used for disease screening, including for human immunodeficiency virus (HIV), malaria, tuberculosis, and hepatitis C, and for epidemiology surveillance in cases such as Ebola [8,10]. Although LFSs have aided faster clinical decisions in many disease screening applications, they cannot perform advanced bioassays to answer more complex biomedical questions [9,10,11,12]. For example, drug resistant types of malarial strains have been diagnosed in large groups of people among African populations, and these malarial strains can easily escape the detection based on the current LFS test, perpetuating life-threatening conditions [13,14].

Further development of the diagnostic tools for EPOCT has been a focus for various research groups in recent years [15]. Revolutionary technologies like microfluidic paper-based analytic devices (µPAD)s [16], multiplexed 2D paper devices [17], and mobile phone-based technologies [18,19] have demonstrated their potential to work effectively in POCT and EPOCT conditions. Regardless of its successes, LFS’s limitations still remain a stumbling block.

Recent publications demonstrate significant interest in the microfluidic research community in exploring the advantages of centrifugal microfluidics for diagnostics in developing countries [20,21,22]. In this paper, we have distinctively captured the infrastructural and technological challenges and the diagnostic needs in EPOCT that can transform centrifugal microfluidics and make it suitable not only for applications in POCT but also EPOCT. This paper provides an overview of the present and future diagnostic needs in extreme settings in Section 2. A general construction and setup of centrifugal microfluidic devices are given in Section 3, followed by a few centrifugal microfluidic based IVD tests according to their biological relevance. Section 4 describes the design challenges for centrifugal microfluidics in different healthcare infrastructures in developing countries, with particular attention to EPOCT.

## 2. Evolving Diagnostic Needs in Resource Limited Settings

To understand the diagnostic needs in resource limited settings, a clear understanding of the types of diseases and their effect on life expectancies is mandatory. The United Nations (UN) and other organizations have been effectively monitoring the disease burden and trying to achieve specific goals that have been set to improve the health of the population in developing countries and negate the imbalance in healthcare—one such initiatives being the UN’s Millennium Developmental Goals (MDGs) [23]. The MDGs have helped to promote the development of new POCT technologies in the last 10–15 years; these new technologies have performed well in challenging environments and have improved life expectancies for people living in developing countries. For instance, the mortality rate of children less than five years old has declined by more than half in Sub-Saharan Africa [24]. Diagnostics was a critical tool for achieving these goals and will remain so for future endeavors in global heath. One of the MDGs concerns combating diseases such as HIV, malaria, and tuberculosis [22]. This initiative has benefited greatly from POCT devices, with LFS assisting in the diagnoses for millions of people in limited resource settings, who could then initiate appropriate treatment at early disease stages, such as antiretroviral (ART) therapy.

**Figure 1:**
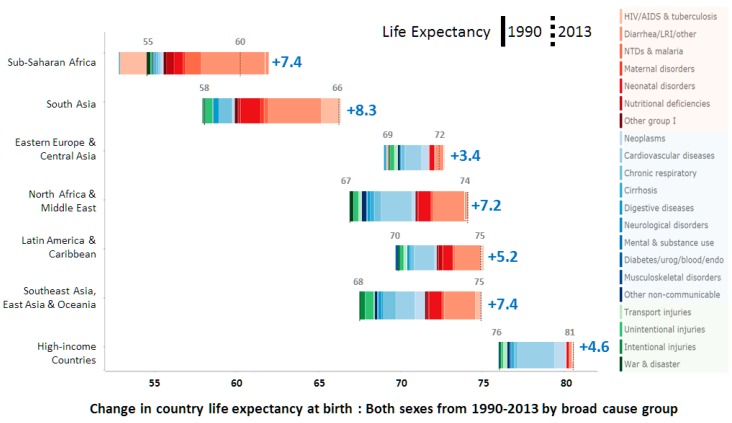
Life expectancies at birth in 1990 and 2013 for both sexes and different causes of life expectancy variation in different regions [26].

Between 1990 and 2013, life expectancy significantly increased in developing countries in Africa, Asia, and Latin America, as depicted in Figure 1. However, Figure 1 also illustrates the existing burden of communicable diseases like HIV, tuberculosis, diarrhea, and other neglected tropical diseases (NTDs), which remain a major cause of death among children and adults [25]. Various studies have shown that early diagnosis using LFS platforms benefits disease prevention and treatment and also lowers the risk of transmission; nonetheless, the lack of effective quantitative tools may lead to new problems in the near future.

Diagnostic tools like LFS have aided this transformation. Even today, LFS is used as the first line of diagnosis, enabling patients to receive immediate treatment [27]. However, current diagnostic needs have evolved. Compromises in diagnosis have resulted in larger complications including medication errors, drug resistant diseases, *etc.* At present, many patients in African and Asian countries contract drug resistant strains of disease causing microbes. This has not only raised the need for new drugs and diagnostic technologies but remains a global epidemic threat [28,29]. For example, viral load testing is an important prognostic tool for patients subjected to ART; this requires a sensitive diagnostic procedure generally performed in a centralized laboratory or hospital. The lack of this type of quantitative diagnosis in remote settings is a huge challenge to rural healthcare efficacy [30].

The UN has announced revised pledges to deliver good health, referred to as the Sustained Developmental Goals (SDGs). Among the SDGs’ many ambitious goals, the key area that can be bridged with EPOCT is to end diseases like AIDS, malaria, tuberculosis, and other NTDs by 2030 [31]. The prevalence of such diseases is very high in extreme settings, and medication errors in these settings is a common difficulty that results in complications like drug resistance resulting in treatment failure. Realistically, improved diagnostic tools are needed in these settings to achieve the SDGs. Affordable technological health interventions like vaccines and drugs have made a significant impact in the past, but new challenges demand precise diagnosis for lower rates of infection. LFSs are used extensively in frontline diagnosis, but diseases that are near elimination require molecular level diagnostics in EPOCT [30]. From Figure 1, it is seen that non-communicable diseases are also increasing in developing countries, which also indicates a need for platform-based technologies. Integrated systems using centrifugal microfluidic technologies are ideally suited to current demands, and effective quantitative EPOCT systems could be delivered if such systems are based on a clear understanding of the end user’s needs and relevant system functions.

### 2.1. Healthcare Infrastructure in Developing Countries

To deliver effective healthcare in developing countries, understanding the disease and diagnostic needs is not sufficient; one also has to have a detailed understanding of the existing healthcare infrastructure to determine the exact extent of resource limitations. The various types of clinical settings in developing countries can be categorized into three major types, as shown in Table 1; urban health centers or hospitals, primary healthcare centers, and non-clinical settings. Each of these types has its own limitations in terms of infrastructure [32], referring to supporting equipment, human resources, and treatment environment in this context.

The extreme settings are comprised of both non-clinical settings (field clinics, mobile health care units, *etc.*) and primary healthcare centers in some cases. Despite the infrastructural challenges between different healthcare settings, the aim for diagnosis remains the same [33]. The unique advantages of centrifugal microfluidic based POCT devices to be used in urban health centers and centralized laboratories have been known [4]. All urban health centers or hospitals and major primary healthcare centers have infrastructure to operate portable POCT devices capable of detecting a disease, either quantitatively or qualitatively, [15]. Cepheid’s Genexpert and Malaria LFS are a few examples of such POCT devices. Table 1 shows that, in non-clinical settings, there is no infrastructure to support any kind of clinical procedure, and the little infrastructure available relies on semi-trained professionals, unreliable electricity, limited or no internet connectivity, and lack of clean environments. Such harsh conditions define the extreme settings that demand EPOCT.

**Figure 2:**
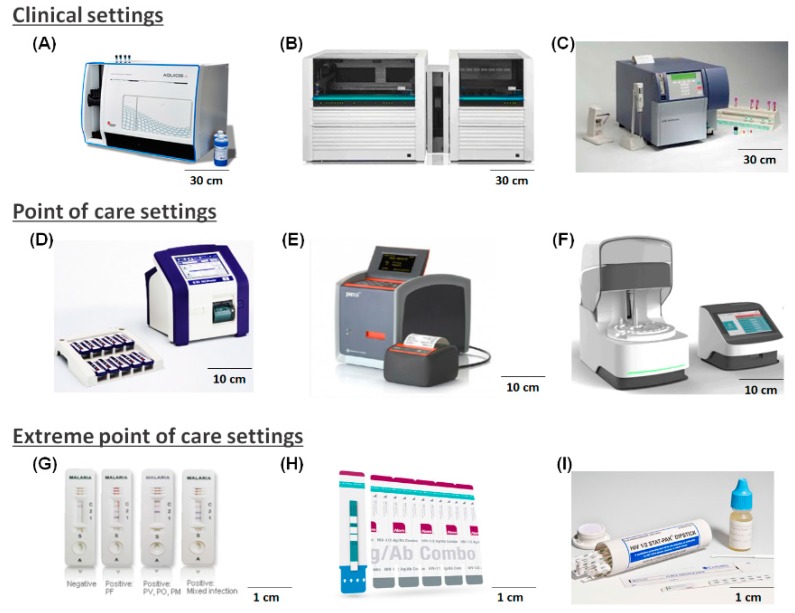
Examples of IVD technology in different treatment settings. Clinical settings: (**A**) Aquios CL flow cytometer (Beckman Coulter, Inc., Brea, CA, USA) for CD-4 testing; (**B**) COBAS^®^ AmpliPrep/COBAS^®^ TaqMan^®^ HIV-1 Test, v2.0, (Roche Diagnostics, Indianapolis, IN, USA); (**C**) BD FACS Count Instrument with Kit (Absolute CD4+, CD8+, and CD3+ Counts), (BD Biosciences, San Jose, CA, USA). POC settings: (**D**) BD FACSPresto^TM^ (BD Biosciences, San Jose, CA, USA); (**E**) Pima CD4 Test (Alere^TM^, Waltham, MA, USA); (**F**) Samba II (DRW (US) Ltd., Sunnyvale, CA, USA), approved in a few African countries, yet to be certified by the World Health Organization (WHO). EPOCT settings: (**G**) CareStart™ Malaria RDT (Access Bio Korea, Inc., Seoul, Korea); (**H**) Alere Determine HIV-1/2 Ag/Ab Combo (Alere^TM^, Waltham MA, USA); (**I**) HIV 1/2 STAT-PAK® dipstick Assay (ChemBio Diagnostics sytems, Inc., Medford, NY, USA) [38,39].

Though EPOCT is not well defined and is an uncommon term, we have distinguished between the two here for a better understanding. In extreme settings, healthcare is accessed through a community health worker who performs testing at a patient’s house or at a temporary or semi-permanent healthcare facility in a remote village or a mobile health unit [32]. Figure 2 shows the differences in the types of devices used in POCT and EPOCT settings based on the available infrastructure in these settings. Recent reports suggest that these suggested diagnostic procedures need to be verified against a gold standard diagnostic procedure before medication is administered, but in reality, this does not occur [35]. Alarming rates of drug resistant strains of malaria, tuberculosis, and mixed infections cannot be diagnosed in extreme settings [28]. Primary healthcare infrastructure has improved in recent years, but has not reached its full potential [36]. Another area of focus in healthcare infrastructure development is healthcare data storage and retrieval using server- or cloud-based systems for screening and monitoring [37].

### 2.2. IVD in Developing Countries

Different kinds of devices are used for IVD in developing countries, depending on the infrastructure in the healthcare setting. Figure 2 shows several examples of diagnostic devices currently approved by the World Health Organization (WHO) for use in developing nations [38,39]. These devices can be laboratory grade devices for clinical settings, relatively small devices for POCT settings, or very simple and easy to use devices for EPOCT settings.

Laboratory grade devices such as the flow cytometer and molecular diagnostic device are available in a centralized laboratory or hospital; these devices can perform complex diagnosis and occupy a large space. These devices also demand specific conditions such as temperature-controlled environments, uninterrupted power, trained operators, *etc.* POCT devices are used in settings with minimal resources and are effective for repetitive diagnostic procedures in specific tests or biological applications in primary healthcare settings. EPOCT settings lack any of the above resources.

Stringent quality criteria specific to each of these settings are regulated by the WHO because almost 68% of the countries in these regions lack any regulations [40]. The WHO periodically releases an updated list of prequalified diagnostic devices once they are tested and approved for use in healthcare settings in developing countries. This quality assurance has helped to maintain good quality in diagnostic products and remove clinically unfit devices [41]. The WHO has prequalified diagnostic procedures for high burden diseases like HIV, malaria, and the hepatitis C virus to the greatest extent possible, and they have also helped in developing diagnostics for NTDs. The WHO evaluate a POCT or EPOCT test using the ASSURED benchmark (affordable, sensitive, specific, user-friendly, rapid and robust, equipment-free and deliverable to end users). Every test used in extreme point of care or resource limited settings is evaluated using this criteria [42].

Figure 2 shows examples of IVD devices arranged according to the healthcare infrastructure. The diagnostic devices on top are capable of handling large sample numbers and a variety of tests are used in clinical settings. The middle row shows the POCT devices used to perform specific tests in primary healthcare settings, and the bottom row shows LFSs used in EPOCT settings. EPOCT cannot be deemed completely successful until certain outstanding challenges have been resolved. For example, in the case of malaria, rapid diagnostic tests (RDT) cannot detect multi-parasite infections [43], and it has been reported that that in South Africa, only 69% of patients with HIV receive periodic laboratory-based CD4 counts [44]. Recent reports have indicated that RDT and LFS may not have the appropriate sensitivity for testing and monitoring certain emerging diseases, and experts have therefore reported the need for alternative technologies that can assure sensitive and selective diagnosis for a particular disease and provide results during a clinic visit or within a reasonable waiting time [33]. POCT devices such as Samba II and Pima CD4 tests are better technologies but are still considered too bulky and ineffective for EPOCT. Several other diseases also require a quantifiable diagnostic technology for EPOCT settings [37].

## 3. Centrifugal Microfluidic-Based IVD

This section focuses on centrifugal microfluidic-based systems for IVD applications. Section 3.1 describes the instrumentation and operation of a commercially available centrifugal microfluidic system, and Section 3.2 discusses clinical applications of the system described in Section 3.1, which has been tested for adaptability in POCT settings and found to be suitable for applications including clinical chemistry, immunoassays, and nucleic acid tests.

### 3.1. Instrumentation and Operation of Centrifugal Microfluidic Systems

Centrifugal microfluidic systems consist of two major components: the disc and the device, generally a single rotor equipped with simple peripherals. The centrifugal microfluidic device uses the same principle of centrifugal force as bulkier centrifuges. This force coupled with the microfluidic technology helps to reduce the sample volume, resulting in a faster reaction time and also enabling elimination of the external pumping sources. Centrifugal pumping is used to move the fluids, which is radially outward from the center, and the movement from one chamber to the other is controlled by microstructures. Rotational speed, connected channel dimensions, location of the fluidic chamber, and viscosity of the fluid determine the flow rate in the channels. Complex microfluidic device operations such as mixing, separation, volume metering, aliquoting, flow switching, and valving are all possible on the single polymer disc, which has embedded within it the complete microfluidic architecture. The disc is fabricated from thermoplastic materials such as polycarbonate, polymethyl methacrylate, or cyclic olefin copolymer; hence, the cost per test can be significantly reduced relative to other IVD techniques [45,46]. Moreover, the transparent optical property of these materials allows direct visualization of the process, and direct optical detection on the disc.

The instrumentation for centrifugal microfluidics includes a rotor with an adaptor that fits the disc, and simple peripherals such as laser diodes, infrared lamps, magnets, and LEDs. These peripherals facilitate advanced operations on the disc, including valve actuation on demand, heating of the disc, manipulation of the magnetic field, and detection of optical signals, to integrate and automate more complex reactions. The automation of assays with these simple instruments render it an ideal candidate for POCT and EPOCT applications.

### 3.2. Clinical Applications

**Figure 3:**
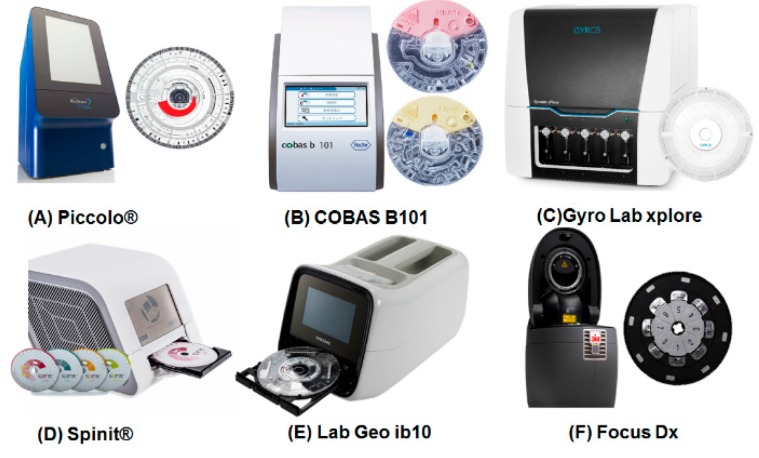
Examples of centrifugal microfluidic-based diagnostic devices for POCT settings: (**A**) Piccolo^®^ by Abaxis (Union City, CA, USA); (**B**) COBAS B101 by Roche Diagnostics (Indianapolis, IN, USA); (**C**) Gyro Lab Explore by Gyros (Uppsala, Sweden); (**D**) Spinit^®^ by Biosurfit (Lisboa, Portugal); (**E**) LAB GEO ib10 from Samsung healthcare (South Korea); (**F**) Focus Dx (Quest) by 3M (Cypress, CA, USA).

IVD can be broadly classified into three areas: clinical chemistry which identifies electrolytes, minerals, and protein concentrations in a given biological fluid; immunoassays which use antibodies to detect disease or disorders; and nucleic acid tests that are used when diagnostics demand very high precision and sensitivity. The commercially available IVD centrifugal microfluidic devices in Figure 3 can perform these complex biological applications in the “sample to answer” manner. These devices can be applied to perform individual applications such as blood chemistry measurements, lipid profiling, virology, *etc*.

#### 3.2.1. Clinical Chemistry

In developing countries, the primary clinical chemistry needs are in the areas of non-communicable diseases among adults, malnutrition among children, and deficiency of vital nutrients during prenatal and maternal care. In response to these needs, Piccolo^®^ from Abaxis is a representative centrifugal microfluidic product that can perform blood chemistry applications. The user can select a specific disc for a particular diagnosis, and the operating system recognizes the disc type by reading barcodes on the disc; the disc is then operated with a spinning profile appropriate for the disc type. This system facilitates a fully automated assay of more than 10 chemicals (various lipid panels and electrolytes) in a blood sample, performing plasma separation, pre-loading of all dried reagents into the disc to reduce user handling, and mixing and detection within 12 min. Therefore, Piccolo^®^ is a good centrifugal microfluidic model for use in IVD as well as POCT. COBAS B101 from Roche Diagnostics further simplifies the sample introduction step for POCT by using blood from a pricked finger. Clinical chemistry involves relatively simpler assays than other IVD tests, and microfluidic operations can be fully automated for the entire assay simply by manipulating the inherent micro-geometry of the disc. This technique has enabled full automation in several commercially available centrifugal microfluidic devices, rendering them the most appropriate devices currently available for POCT.

#### 3.2.2. Immunoassays

Immunoassay is the most well-known method of IVD; this technique is used to detect protein-based biomarkers from biofluids such as saliva, blood, or urine. Immunoassays are used to detect both communicable and non-communicable diseases, and are therefore in high demand in developing countries. Immunoassays involve several sequential steps, including blood separation, target and antibody incubation, washing, and detection. Therefore, robust and accurate microfluidic flow control is required for full integration. The company Gyros has provided first generation commercialized centrifugal microfluidic devices for immunoassay applications. In the Gyros devices, a hydrophobically treated region enables sequential flow control, and an injection-molded disc allows exact metering of reagents to reduce error. One disc contains 112 channels, making the device attractive for high throughput immunoassays. However, this device is considered to be more appropriate for use in a centralized laboratory because it does not perform plasma separation and because a delicate robotic liquid handler system is required to introduce the reagents. Samsung Electronics and Biosurfit provide more appropriate centrifugal microfluidic devices for immunoassays in POCT settings. In these devices, once blood is injected into a disc, the operating system performs an automated immunoassay and detects the result a few minutes later. The Samsung and Biosurfit systems are designed to detect cardiac protein biomarkers; however, they can be repurposed for the detection of other proteins, as is needed in developing countries.

#### 3.2.3. Nucleic Acid Tests

Nucleic acid tests facilitate the detection of a target molecule at the molecular level and have thus become an important method for the detection of many diseases. Compared to the other two IVD techniques, full integration of nucleic acid tests in commercially available centrifugal microfluidic devices remains challenging. It requires a robust and precise microfluidic network because the detection of nucleic acids often requires a large sample volume and the assay consists of a complex series of steps, including sample concentration, nucleic acid extraction, target purification, amplification, and detection. In addition, a precise and accurate heating source is a necessary system component because the amplification step requires preventing reagent evaporation. For this reason, no centrifugal microfluidic device has yet integrated the entire nucleic acid detection process. Toward this objective, Focus Diagnostics (Cypress, CA, USA) provides a universal disc for nucleic acid amplification and detection. In addition, fast and accurate thermocycling for the amplification step can be achieved with an infrared heater and disc body rotation for effective cooling. Moreover, a few companies are currently preparing the products for simplified nucleic acid analysis [47,48].

## 4. Centrifugal Microfluidic Systems for EPOCT

### 4.1. Opportunities of Cenfrifugal Microfluidics for EPOCT

Design and development of diagnostic devices for use at the extreme point of care is not the same as compared to clinical point-of-care. For an extended time period, the diagnostic technologies developed for use in developing countries were mostly a modified of existing diagnostic technologies in use in the developed world. From our understanding of healthcare systems in resource limited settings and insights gleaned from recent reports on diagnosis in extreme settings, we believe an inclusive understanding is required to rightly distinguish POCT from EPOCT needs. Healthcare infrastructure-dependent requirements for biomedical devices are described in Table 2. Most of lab-on-a-chip can meet several requirements such as low sample volume, easy operation, and fast process time. Above all, centrifugal microfluidics can be distinguished with other platforms since they enable a real sample-to-answer system for the user including the sample purifications. Many biological samples like saliva, urine, and blood need sample purification before the utilization for assays. It is well known that biofluids contain many inhibitors that can cause inaccurate assay results. The simplest method of sample purification is centrifugal force induced separation and most of the chip based microfluidics studies rely on centrifugation of samples before the introduction to their chip. However, this crucial step in the process can be easily be integrated onto centrifugal microfluidic system, and it allows for the realization of a real “sample-to-answer” system for the end user. It has already been validated that sample preparation for various fluids can be integrated on centrifugal microfluidic systems [49,50,51,52,53]. Nevertheless, to the best of our knowledge, there are no centrifugal microfluidic devices currently available for use in EPOCT settings. Previous reports of lab-on-a-chip and LFS devices were used to formulate the EPOCT requirements shown in Table 2 [34,37,54]. Technological developments required for the realization of centrifugal microfluidic devices that can be used in EPOCT conditions and its supporting instruments are explained in next section.

### 4.2. Challenges of Cenfrifugal Microfluidics for EPOCT

#### 4.2.1. Disc Requirements

EPOCT settings have no infrastructure to support clinical grade diagnosis, and the disc must therefore be designed to meet these unique EPOCT demands. Centrifugal microfluidic discs are commonly described as fully integrated; however, a fully integrated disc to be used in EPOCT settings should have different characteristics as mentioned in Table 2. In EPOCT settings, the cost per test is a major challenge; in a disc, there are two cost factors, the disc and the biological reagents used for the assay. Novel cost effective fabrication methods have been reported in recent years and it is advisable to apply the best possible solution to have the lowest possible cost per test. Developing an inexpensive disc manufacturing technique would further reduce the overall cost of the system. Recent reports on inexpensive fabrication techniques appear promising [48,49]; however, the fabrication of electrodes, valves, heating elements, *etc.*, on the disc can increase the cost of the device, and large scale fabrication of fully functional discs at minimal cost remains as a challenge.

In POCT settings, diagnostic procedures are generally performed by healthcare workers, whereas in EPOCT settings, fewer personnel with significantly less training are deployed to implement care. Therefore, EPOCT systems need to be a foolproof system that functions like a ‘sample in answer out’ system, which does not require any manual execution of intermediate steps. This is ideal in EPOCT settings. In extreme settings, the samples are limited to saliva, urine, nasal fluid, tear drops, and blood (by finger prick and not by syringe) to avoid complication and contaminations; therefore variation in the samples is common and field samples must be used to standardize the testing procedure. Recent reports show the sensitivity and specificity of the test needs to be evaluated as clinical decisions are made based on test results [33]. Because of the low sample volumes in EPOCT settings, an appropriately simplified sample collection system, specific to each biological fluid, should be integrated in the diagnostic system. For example, a capillary based sample loading inlet on a disc would be ideal for collecting blood samples, direct swab inlet ports on disc for saliva samples will make a difference.

Temperature variations have been constantly reported as the major challenge as it directly affects the sensitivity of reagents and sensing elements, and therefore proper precautions must be taken when selecting the biological reagents and sensing elements for EPOCT settings. For example, aptamers can be used as stable sensing elements, and similarly, enzymatic reagents can be lyophilized. Lack of proper waste management in EPOCT settings are another concern, and hence the waste chamber in the disc should be filled with disinfectant to prevent the spread of disease. Lastly, the discs must be able to be carried conveniently in large numbers so they can be transported along with the device.

#### 4.2.2. Supporting Instruments

Although the simplicity of centrifugal instruments make them ideal candidates for POCT, only with certain revisions can they be implemented as a successful EPOCT device. EPOCT settings place unique demands on the instruments in areas of cost, power, data storage and retrieval, design, robustness and low maintained. Affordability is a broad term used for specifying the cost of the instrument in most of the literature. Instruments like centrifugal microfluidic devices which can perform a range of diagnosis have a good cost *vs.* benefit ratio. From recent recommendations for HIV diagnosis, the cost of the instruments stays affordable when it is less than US$ 500 per machine [55]. Most of the EPOCT settings lack uninterrupted power supply and hence the instrumentation design should be such that it can be operated without an external power source or solar powered. The supporting instruments should consist of minimal components, as these devices will not only be transported to remote locations, but also because minimal components will result in a user friendly design that will assist the end user, who will usually not be highly trained. Along with ease of transport, durability is another major requirement of EPOCT designs. Hand-held devices and others with minimal footprints and compact systems would be ideal for use in EPOCT settings. The instrumentation should be self-sustaining in terms of power because of the lack of reliable power supplies in EPOCT settings; therefore, devices that are battery operated and consume little power are preferred. The total turnaround time from sample to answer should also be as low as possible, which is a primary reason for the popularity of LFS and RDT techniques. Furthermore, because the device must be transported, simple built-in detectors should be used in centrifugal microfluidic systems, such as has recently been reported with the use of DVD and Blu-ray lasers for the detection purpose.

The overall strategy for developing an EPOCT centrifugal microfluidic device relies on a medium to low cost device with a low cost disc that can store reagent and is fully integrated. The detection technique should be selected to leverage the use of the instrument for the widest possible range of biological applications. Electrochemical detection and absorbance offer low power, low cost, and high sensitivity detection methods, well suited to the demand. The lack of trained professionals in EPOCT settings demands a fully automated sample-to-answer system with a user friendly interface. Once the sample is inserted, an automated spinning profile should be able to sequentially process the sample and provide a readout. The disposal of the processed sample should be safe, which can be achieved by storing the waste inside the disc to prevent contamination due to handling [34]. Improvements in health information technology have enabled better data connectivity in these systems, and therefore the device should have network connection capability, either directly or via a mobile phone interfaced with the device [30]. Lastly, it has recently been reported that collaborating with local partners and adopting a patient-centered approach will yield more successful functionality of system designs [15].

Even though a handful of centrifugal technologies are available with the POCT platform, there are no centrifugal microfluidic devices currently available for use in EPOCT settings. As distinguished and defined in this paper, EPOCT conditions will bring new challenges, both for researchers and companies who have interest in these segments. As illustrated, centrifugal microfluidics has shown advantages over other microfluidics and LFs technology. The advantages like cost, compactness, complexity free and the wide spectrum of biological techniques that can be performed positions centrifugal microfluidic systems as forerunners in the race to meet challenging EPOCT requirements As advantageous as it is in its current form, it still meets the requirements for a perfect EPOCT and needs improvement in areas that we have discussed above. We have discussed the suggestions as preliminary directions for improvement; as demands evolve; the technology should be able to able to bridge the gap and meet EPOCT needs.

## 5. Conclusions

A review of centrifugal microfluidic systems for use in EPOCT settings has been presented, and the evolving diagnostic needs of developing countries have been discussed. Various centrifugal microfluidic devices that have been developed and commercialized were discussed and the key considerations for designing and evaluating EPOCT centrifugal microfluidic devices have also been presented. As identified in this review, diagnostic devices designed for low and middle income countries require a different approach than is applied to standard clinical practice. The centrifugal microfluidic systems currently used in POCT settings cannot be directly incorporated into EPOCT settings. Based on these recommendations, the unique requirements of EPOCT settings can be integrated onto the centrifugal microfluidic disc and the supporting instrumentation. The challenges facing EPOCT are significant, and the opportunities offered by centrifugal microfluidic diagnostic devices are therefore considerable. It can thus be stated that, if the above mentioned recommendations were to be considered and integrated into existing centrifugal microfluidic devices, they could be made ready to use in EPOCT settings.

## Figures and Tables

**Table 1:** Clinical infrastructure in developing countries in Asia (South Asia, East Asia, Southeast Asia, Oceania); Africa (Sub-Saharan Africa, North Africa) and Latin America [32,33,34].

Infrastructure Availability	Urban Health Centers or Hospitals (59%)	Primary Healthcare Centers (27%)	Non-Clinical Settings (14%)
Clean water	O	×	×
Electricity	O	Δ	×
Dust free environment	O	Δ	×
Cold storage	O	×	×
Stable temperature	O	×	×
Trained professional	O	O	×
Internet access	O	Δ	×

Key: available (O), partially available (Δ), unavailable (×). Urban health centers or hospitals are usually established by the state or the central government and perform most diagnostic testing in their respective countries. Primary healthcare centers are found in rural areas, and are often connected to the urban hospital, and are minimally equipped, including, for instance, a microscope for cell counting, a manual or electric centrifuge, LFSs and reagents for other colorimetric tests. Non-clinical settings can be a patient’s home or a gathering in the common area of a village or other rural locations where a test is performed.

**Table 2:** Healthcare infrastructure-dependent requirements for biomedical devices [34].

Healthcare Setting	Urban Health Centers or Hospitals	Primary Healthcare Centers	Non-Clinical Settings
Device used	Non-POCT	POCT	EPOCT
Sample type	All kinds of samples (Venous blood, saliva, sputum, urine, nasal fluid, *etc*.)	Minimally invasive samples (Finger prick blood, urine, saliva, *etc*.)	Minimally invasive samples (Finger prick blood, urine, saliva, *etc*.)
Sample volume	High (>1 mL Blood)	Low (<10 µL Blood)	Low (<10 µL Blood)
Sample preparation	Manual/Automatic	Semi-automatic	Automatic
Sampling Size	Many	One ~ Few	One
Existing laboratory equipment	Advanced	Simple (Centrifuge, light microscope, *etc*.)	None
Device footprint	Large	Medium	Hand-held
Power supply	Normal	Battery/normal	Battery
Operation difficulty	Low ~ High	Medium	Low
Processing time	Fast/moderate	Fast	Fast
Usage	Heavily used	Minimally used	Used on demand
Durability	Low	Medium	High
Device cost	High	Medium	Low
Consumable cost	Low ~ High	Low	Low
Diagnostics	Screening, Quantification	Screening, Quantification	Screening, Quantification
Medical data storage	Local, Cloud	Local, Cloud	Cloud
